# Analysis of the Retinal Nerve Fiber Layer in Retinitis Pigmentosa Using Optic Coherence Tomography

**DOI:** 10.1155/2015/157365

**Published:** 2015-08-16

**Authors:** Medine Aslı Yıldırım, Burak Erden, Mehmet Tetikoğlu, Özlem Kuru, Mustafa Elçioğlu

**Affiliations:** ^1^Department of Ophthalmology, Bahcelievler State Hospital, 34180 Istanbul, Turkey; ^2^Department of Ophthalmology, Okmeydanı Education and Research Hospital, 34384 Istanbul, Turkey; ^3^Department of Ophthalmology, Dumlupinar University School of Medicine, 43270 Kutahya, Turkey; ^4^Department of Ophthalmology, Mus State Hospital, 49000 Mus, Turkey

## Abstract

*Aim*. To evaluate the peripapillary retinal nerve fiber layer (RNFL) changes in retinitis pigmentosa (RP) patients using spectral domain optic coherence tomography (Sd-OCT). *Methods*. We retrospectively examined medical records of forty-four eyes of twenty-two RP patients. The results were also compared with those of previously reported forty-four eyes of twenty-two normal subjects (controls). Records of average and four quadrants peripapillary RNFL thickness measurements using Sd-OCT were assessed. *Results*. In RP patients the mean RNFL thickness was 97.57 ± 3.21 *μ*m. The RNFL in the superior, temporal, nasal, and inferior quadrants was 119.18 ± 4.47 *μ*m, 84.68 ± 2.31 *μ*m, 75.09 ± 3.34 *μ*m, and 113.88 ± 4.25 *μ*m, respectively. While the thinning of RNFL was predominantly observed in the inferior quadrant, the thickening was mostly noted in temporal quadrant. The differences between mean, superior, and nasal quadrant RNFL thicknesses were not statistically significant when compared with control group. The RP patients had thinner inferior quadrant and thicker temporal quadrant than control group (*p* < 0.05). *Conclusion*. Sd-OCT is highly sensitive and effective instrument to detect RNFL changes in RP patients. RNFL measurements can provide information about the progression of retinitis pigmentosa and may provide prognostic indices for future treatment modalities.

## 1. Introduction

Retinitis pigmentosa (RP) is a genetically heterogeneous disease characterised by progressive retinal photoreceptor degeneration [1, 2]. The worldwide population affected has been estimated to be over one million individuals, whereas the frequency is approximately 1/4000 [3, 4].

Although RP has several mutations and genetical patterns, the symptoms and histopathological findings are similar [5]. Common symptoms are nyctalopia, impairment of visual acuity, and restriction of peripheral visual field. Characteristic findings by fundus examination include peripheral pigmented bone spicule-like lesions, retinal arteriolar attenuation, and optic disc pallor [6]. Diagnosis is often made through a combination of clinical investigation, visual field exams, and electrodiagnostic methods such as electroretinography (ERG) [5].

Various histopathological studies of RP have demonstrated a reduction of rod and cone cells and thinning of the outer photoreceptor layer. Secondary to the outer retinal thinning, the inner retinal structure degenerates through suspected transneuronal damage, vascular compromise, or axonal compression [7–9]. Previously published reports by Newman et al. demonstrated that patients of various retinal hereditary dystrophies, including RP, had ophthalmoscopically evident retinal nerve fiber defects [10].

The optic coherence tomography (OCT) is a noninvasive diagnostic tool for rapid scanning and imaging of the retinal structures with high axial resolution of almost 5 *μ*m, especially in spectral domain models. The OCT scans give the clinician crucial information about the retinal nerve fiber layer (RNFL), retinal pigment epithelium complex, and the junction of inner and outer segments of photoreceptors (IS-OS line). The new therapeutic modalities in RP, such as gene therapy [11–14] or retinal stem cell transplantation [15, 16], are limited because they only improve the outer retinal layers. The outcome of new therapies must focus on the pretreatment status of the inner retinal layers as well as the outer layers making OCT a useful technique in the treatment prediction for such RP patients. In this study, we aimed to examine the peripapillary RNFL thickness and documented the changes in a group of RP patients.

## 2. Methods

Medical records of forty-four eyes of twenty-two RP patients who were followed up in Okmeydanı Training and Research Hospital, Department of Retina, and forty-four eyes of twenty-two healthy subjects (control) were enrolled into this study. The diagnosis in all cases was made by retinal specialists using the following criteria: clinical history, fundus examination, visual field defects, and reduced amplitudes in ERG.

The exclusion criteria used in our study were high refractive errors (±6 diopters sphere; ±3 diopters cylinder), low degree of central fixation, significant media opacities (e.g., posterior subcapsular cataract), diabetic retinopathy, glaucoma, or cystoid macular edema.

All cases underwent a complete ocular examination, including best-corrected visual acuity (BCVA) using Snellen chart, slit lamp biomicroscopic examination, dilated fundus examination, and intraocular pressure measurement with Goldmann applanation.

**Figure 1:**
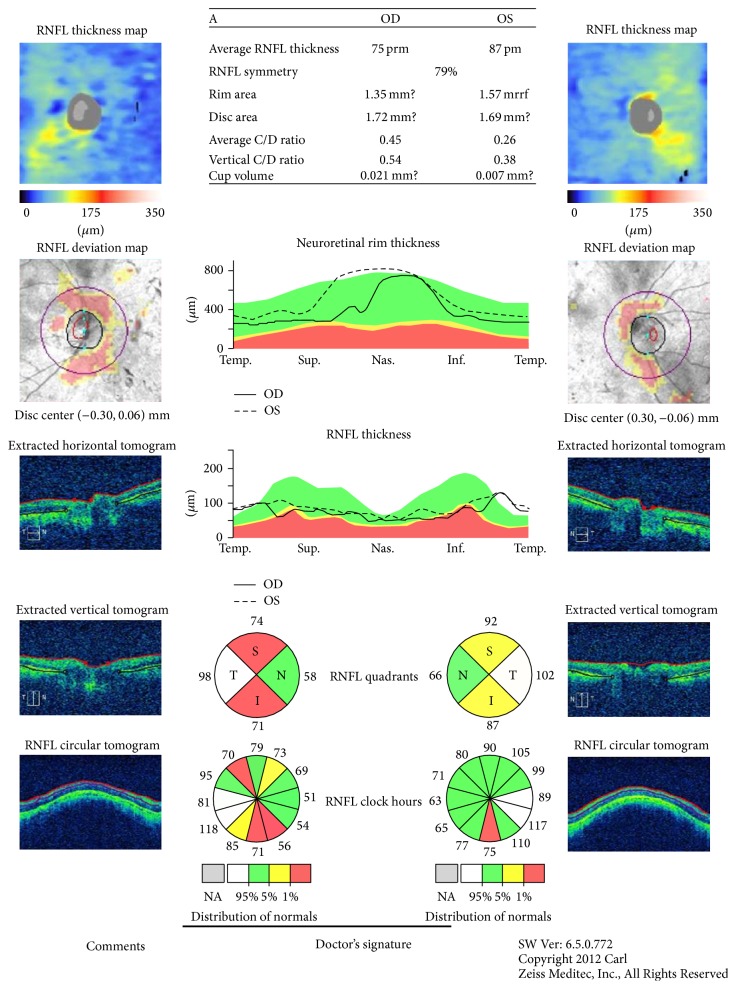
Retinal nerve fiber layer analysis in patients with retinitis pigmentosa.

Average and four quadrants peripapillary RNFL thickness values which were scanned by spectral-domain OCT (Cirrus, Carl Zeiss Meditec, Inc., software 5.1.1.6) made by the same operator were analyzed. The scans only with signal strength > 5 were included in this study. Any RNFL layer thickness greater than 95th percentile was defined as thickening, whereas thickness lower than the 5th percentile was determined as thinning (Figure 1). Statistical analysis of the data was determined with the SPSS 19.0 software.

## 3. Results

The mean age of the RP patients was 37.09 ± 2.55 years (range 18–75) and the mean age of control group was 39 ± 1.70 years (range 18–54). The mean age, sex, and intraocular pressure of the two groups did not differ significantly. In the RP patients the peripapillary RNFL was evaluated and the mean thickness was observed to be 97.57 ± 3.2 *μ*m. RNFL thickness in the 4 quadrants (superior, temporal, nasal, and inferior) was shown to be 119.18 ± 4.47 *μ*m, 84.68 ± 2.31 *μ*m, 75.09 ± 3.34, and 113.88 ± 4.25 *μ*m, respectively.

In 20 of the total 44 eyes (45%), RNFL thinning was found in at least one quadrant. In two eyes (4%) thinning was determined in 3 quadrants, in 11 cases (25%) in 2 quadrants, and in 7 eyes (15%) in only one quadrant. Regarding the RNFL thinning of quadrants, the thinning was diagnosed in 12 cases (27%) in superior quadrant, in 9 eyes (20%) in the nasal quadrant, and in 14 eyes (32%) in inferior quadrant. Temporal quadrant thinning was not found in any of the patients.

In 21 (48%) patients, thickening of the RNFL was found in at least one quadrant. Five eyes (11%) showed thickening in 3 quadrants, 2 (4%) eyes in 2 quadrants, and 14 (31%) eyes in only one quadrant. Five patients (11%) had thickness in the superior quadrant, 8 patients (18%) were in the nasal, 19 eyes (43%) were in the temporal, and only 1 patient (2%) was in the inferior quadrant. In 8 patients, all 4 quadrants were found within the normal values. In 5 patients there was thinning and thickening of the RNFL layer in different quadrants.

The thinning of the RNFL was most commonly observed in the inferior and moderately in the nasal and superior quadrants. Temporal RNFL thinning was not determined in any patient, whereas the temporal region was the most commonly thickened RNFL area followed by the nasal, the superior, and finally the inferior quadrant.

**Figure 2:**
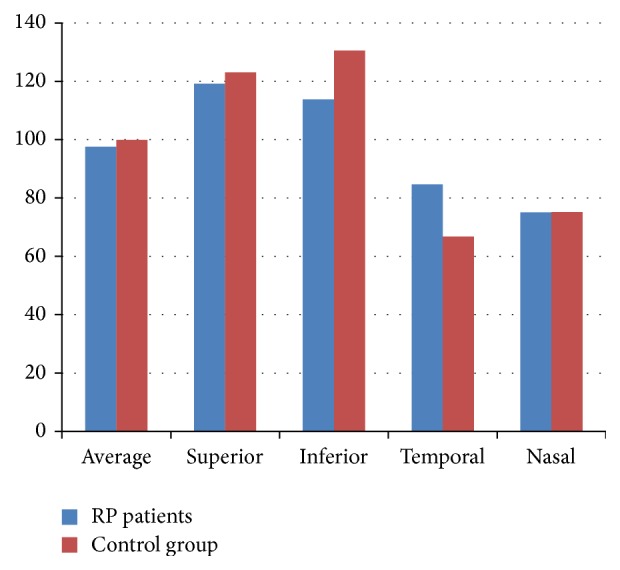
Distribution of RNFL thickness between RP patients and control group.

In control group the average RNFL thickness was 99.95 ± 1.38 *μ*m. In the quadrant evaluation, the RNFL thickness in the superior, temporal, nasal, and inferior quadrants was 123.13 ± 2.96 *μ*m, 66.75 ± 2.02 *μ*m, 75.20 ± 1.44 *μ*m, and 130.54 ± 1.72 *μ*m, respectively. There was no significant difference in average, superior, and nasal thickness between two groups (*p* = 0.497; 0.463; 0.975, resp.). The RP patients had thinner inferior quadrant and thicker temporal quadrant than control group. This difference was statistically significant (*p* = 0.01, *p* = 0.00). Distribution of RNFL thickness between RP patients and control group is shown in Figure 2.

## 4. Discussion

In RP, the photoreceptor layer progressively degenerates, followed by global changes within the inner retinal structure. In particular, the RNFL develops thinning or thickening secondary to photoreceptor cell loss. Morphometric and histological studies reported ganglion cell reduction in RP compared to the normal population [10, 17, 18]. Other reports suggested that vascular compromise or direct genetic effect on the ganglion cells was the reason for inner retinal layer reduction [18, 19].

Regarding the in vivo studies, Walia et al. reported using time-domain and Fourier-domain OCT to define RNFL thinning in 40% and 38% of RP patients [20, 21]. A similar result was reported by Anastasakis et al. [22] which showed RNFL thinning in 38% of studied eyes, whereas Oishi et al. [23] found no significant difference between normal population and RP patient RNFL thickness using time-domain OCT. In our study, 45% of eyes showed RNFL thinning, similar to Walia and Anastasakis' study results. In the previously mentioned studies, the RNFL thickening was found in 40% and 42% of study cohorts, respectively. In our study we found that 48% of the studied eyes had peripapillary RNFL thickening, mostly in temporal quadrant. The exact mechanism underlying this RNFL thickening is not clear but it may result from glial tissue proliferation (which is secondary to the nerve fiber layer atrophy) or edema of the remnant RNFL [23, 24]. In fact, Hood et al. measured RNFL thickness in RP patients from peripheral retina and macular regions with Fourier-domain OCT and manual segmentation software. They found that the RNFL was significantly thicker, especially in the horizontal meridian compared to the normal values [25]. In the same study, it was also suggested that the thickening might be a mechanical dysfunction, where the RNFL stretches to fill the empty space left by the photoreceptor degeneration. The peripapillary RNFL changes in the four quadrants vary between several studies. In histopathological examination of RP patients, Flannery et al. [26] found that the highest ratio of the photoreceptor loss was encountered in the inferonasal region, which may facilitate the RNFL thinning in this region. Walia et al. reported in their studies [20, 21] that the nasal quadrant was the most thinned, followed by inferior and superior quadrants. Anastasakis et al. [22]. observed the quadrants' thinning sequence as inferior (most common) followed by the nasal and superior regions. In both Xue's et al. [27] and Hood's et al. [25] studies, the only thinning quadrant was the nasal area. Xue et al. [27] found that all quadrants in their RP patient study were significantly thicker than the normal population, except the nasal quadrant, which was surprisingly thinner than the normal control group values. In our studies, the mostly thinned quadrant was the inferior followed by the superior and the nasal quadrants. Regarding the thickening of the RNFL, in several studies [20–22, 24, 27], the most commonly thickened quadrant was the temporal. Interestingly, no reports have observed thinning in temporal peripapillary area. Similar to these reports, we found that the temporal quadrant was the most commonly thickened region with no observed RNFL thinning. This might be due to the retinal glial proliferation, which is most strongly seen between the temporal arcades [28].

Considering future therapeutic approaches, understanding the mechanism and progression of retinal degeneration in RP will be crucial to predicting proper patient treatment. High resolution Sd-OCT scans will be extremely helpful for the clinician in both aspects. Further studies might enhance our clinical evaluation of such patients.
